# Prevalence and risk factors for diabetic foot complications among people living with diabetes in Harare, Zimbabwe: a cross-sectional study

**DOI:** 10.1186/s12889-023-17610-7

**Published:** 2024-03-04

**Authors:** Oppah Kuguyo, Doreen Macherera Mukona, Vasco Chikwasha, Lovemore Gwanzura, Joconiah Chirenda, Alice Matimba

**Affiliations:** 1 Department of Clinical Pharmacology, Faculty of Medicine and Health Sciences, University of Zimbabwe, Mazowe Street, Harare, Zimbabwe; 2 Department of Primary Care Sciences, Faculty of Medicine and Health Sciences, University of Zimbabwe, Harare, Zimbabwe; 3 Department of Community Medicine, Faculty of Medicine and Health Sciences, University of Zimbabwe, Harare, Zimbabwe; 4 Department of Medical Laboratory Sciences, Faculty of Medicine and Health Sciences, University of Zimbabwe, Harare, Zimbabwe

**Keywords:** Diabetes, Diabetic foot, Diabetic foot in Zimbabwe, Peripheral neuropathy, Insulin

## Abstract

**Background:**

Diabetic foot disease (DF) is a common diabetes-related complication; however, the prevalence and associated risk factors for DF are not well characterised among people living with diabetes (PLWD) in Zimbabwe. This may suggest the unavailability of adequate strategies to diagnose and treat DF in the country. This study aimed to determine the prevalence of DF and associated risk factors for PLWD in Harare, Zimbabwe.

**Methods:**

This was a cross-sectional study, employing a quantitative approach. In total, 352 PLWD were recruited from 16 primary care clinics in Harare. Sociodemographic and clinical data were collected via face-to-face interviews and clinical records reviews. The DF screening included an evaluation for peripheral neuropathy, ankle-brachial index (ABI), ulceration, and amputation. Self-administered questionnaires were used to assess knowledge, attitudes, and practices (KAPs), and KAP was scored using Bloom’s cut-off. Chi-Square goodness-of-fit tests were performed, and regression analyses were used for association analysis. The threshold for significance was *p* < 0.05.

**Results:**

This group included 82 men and 279 women, with a combined mean age of 57.9 ± 14 years. Twenty one (~ 26%) men and 41 (15%) women had type 1 diabetes. The diabetes type distribution significantly differed by gender (*p* < 0.001). Oral hypoglycaemics (71%) were most commonly administered for management. DF was observed in 53% (95% CI = 50–56) of PLWD. Other DF symptoms observed were abnormal ABI (53%), peripheral neuropathy (53%), foot ulceration (17%) and amputation (3%). Peripheral neuropathy increased the risk of ulceration (OR = 1.7; 95% CI = 1.1–2.6; p = 0.019), while insulin use was protective against amputation (OR = 0.1; 95% CI = 0.1–0.9; *p* = 0.049). Most (87%) of the participants demonstrated good DF knowledge and the importance of adhering to medication to prevent DF. However, 96% did not know that smoking was a risk factor for DF. Nearly two-thirds (63%) demonstrated poor attitudes and practices. Poor attitudes and practices were not predictors of DF ulceration risk (*p* > 0.05).

**Conclusion:**

This study showed that there was a high prevalence of DF (53%) in PLWD in Zimbabwe, and insulin use was protective against DF. There is an urgent need for policy revisions to include foot screening in routine primary care and increasing insulin use for PLWD to prevent complications such as DF as an integral part of primary care.

## Highlights

Diabetic foot (DF) complications can lead to amputations, yet foot care is not routinely monitored in Zimbabwe. Our study describes the DF burden and the associated risk factors among people living with diabetes (PLWD) in Zimbabwe. More than half had DF, and 3% had amputated lower limbs. Insulin use was found to protect against amputations. Our study highlights the need for routine DF screening for PLWD. Our findings also underscore the need for education and awareness tools for patients to adhere to prescribed insulin for improved diabetes management and prevention of complications such as DF.

## Introduction

Diabetes is one of the leading causes of hospitalisations and death worldwide [1]. Diabetes has severe financial implications, especially in developing countries where high comorbidity levels are coupled with constrained manpower and rampant poverty [1, 2]. The prevalence of diabetes continues to rise in Zimbabwe, and in 2018, approximately 850,000 people, or 5.7% of the total Zimbabwean population, were estimated to be living with diabetes [2]. The estimated cost of treatment for people living with diabetes (PLWD) in Zimbabwe is approximately US$1300 per year per patient, while care for diabetes-related complications such as diabetic foot (DF) bears a cost burden of US$2884 per annum [3]. These high costs are a major barrier to adherence to prescribed care in Zimbabwe, where health care is an out-of-pocket expense and there is high unemployment [4]. National health insurance subscriptions are low in Zimbabwe, and there are limited government aid schemes for chronic illnesses such as diabetes [3]. Moreover, this high-cost burden translates to delayed treatment and increased mortality risk – posing a significant public health threat [3, 4].

DF affects 6% of PLWD and encompasses lower extremity complications such as peripheral neuropathy, peripheral arterial disease (PAD), soft tissue infection, and ulceration [5]. More than one-third of PLWD develop DF during their lifetime [6]. The most common DF complication is foot ulceration, which constitutes 25% of all DF complications [6]. Foot ulcerations are associated with the onset of more than 85% of all amputations, and an estimated one in five individuals who develop DF require amputation [6, 7]. Moreover, DF complications pose a great threat to the quality of life for PLWD and also increase the risk of diabetes-related mortality by 2.5-fold [6, 7].

Risk factors for DF include older age, low education, low socioeconomic status, alcohol consumption, smoking, high body mass index (BMI), type of diabetes, poor blood circulation, cardiovascular disease, nephropathy, retinopathy, and claudication [8]. Moreover, good foot care has been shown to prevent 50–80% of DF complications, so good knowledge, attitudes and practices are key to preventing DF [9–11]. Similarly, poor knowledge and attitudes toward DF also translate to poor foot care practices and increase the risk of developing DF [12]. Evidence from studies in diverse socioeconomic settings shows that the prominence of risk differs between populations, due to context-related factors. Therefore, there is a need to conduct research in diverse socioeconomic settings to identify specific local risk factors for DF.

Several studies have investigated DF in Zimbabwe and a wide-ranging prevalence of 1–33% has been reported [13–16]. A longer duration of diabetes, absence of pedal pulses, and peripheral neuropathy were found to be risk factors for foot ulceration in these populations [17]. There are also very few studies that analyse the knowledge, attitudes and practices related to DF in Zimbabwe. However, these studies are few, have small sample sizes, and have focused on single institutions.

The Zimbabwe Diabetic Foot Project (ZDFP) was established to build the capacity for DF prevention and management of DF in Zimbabwe. As a first step, the project sought to use a multicentre approach to understand the burden of DF in Harare, the capital city of Zimbabwe; to understand the context-specific risk factors for DF in Harare; and to build the capacity of nurses at the forefront of managing PLWD, as previously described [18, 19]. The current paper describes the prevalence of DF, and associated risk factors among PLWD attending public health clinics across Harare.

## Methods

### Ethical approval

Ethical approval for the project was granted by the national ethics committee, the Medical Research Council of Zimbabwe (approval number: MRCZ/A/1923). All study protocols were in accordance with the Declaration of Helsinki, 2013.

### Study design and setting

The study utilised a quantitative approach. A cross-sectional study design was used to recruit participants from 16 outpatient diabetes treatment facilities in the public sector, located in Harare. The treatment facilities included 14 primary care clinics and 2 referral hospitals that were conveniently selected because of the availability of registered general nurses at the forefront of managing PLWD, and who had received diabetic foot screening training as part of the ZDFP [18, 19]. Data to assess the knowledge, attitudes, and practices related to DF risk were also collected for analysis.

### Participant recruitment

A consecutive sample of 352 participants was recruited between February 2015 and February 2016. To be considered eligible for recruitment into the cohort, participants had to (i) have a confirmed diagnosis of diabetes, (ii) be aged older than 18 years, (iii) be seeking medical attention for diabetes at the targeted outpatient facilities, and (iv) provide consent to participate. Pregnant women and individuals admitted for inpatient care were excluded from the study. Informed consent was obtained from all study participants. All participants were informed of the study verbally and provided written consent to indicate agreement to participate. All participants were assigned a unique identifier that was used throughout the study to maintain confidentiality.

### Data collection

The data were collected through face-to-face interviews, retrospective patient records reviews, and foot screenings. All the data were entered into a standardised case report form to ensure uniform data capture. Deidentified case report forms were stored in a lockable cupboard with limited access and electronic back-ups were stored on a secure laptop.

#### Face-to-face interviews

The participants provided data on age, sex, literacy status, socioeconomic status, employment status, and behavioural factors such as smoking habits, and alcohol consumption history. Data on family history of diabetes and cardiovascular disease was also recorded.

#### Patient record reviews

Specific data related to diabetes, including body mass index (BMI), type of diabetes treatment, duration of diabetes, comorbid conditions, status and history of hypertension, coronary heart disease, cerebrovascular stroke, claudication, revascularization, renal transplantation, dialysis, or laser photocoagulation were collected from the patients’ clinical records. In addition, a history of amputation, foot infection, nephropathy, retinopathy, and symptoms of diabetes-related neuropathy data were also collected.

### Diabetic foot screening

Diabetic foot screening was performed by two registered general nurses who were trained under the ZDFP to conduct comprehensive foot screening for DF as previously described [18, 19].

#### General foot assessment

A general foot examination for the presence of discoloured toenails, calluses, fissures, corns, and interdigital infection, the condition of the toenails and toe hair, and joint flexibility, was performed. A visual inspection for active foot ulceration or healed ulcer wounds as well as amputation of the toes or limbs was also conducted as a marker for diabetic foot ulceration (DFU).

#### Sole pressure assessment

Deformities that may result in repetitive and excessive pressure on the foot, including hallux valgus, pes planus (flat foot), pes cavus (high arch), hammer toe, and Charcot joint were assessed via visual inspection by the registered general nurse. Confirmation of the presence of these conditions was made by a foot imprint captured on a Foot Imprinter Harris Mat FM1111 (Diabetik Foot Care India Pvt Ltd, India), which was shown to a podiatrist for accurate determination.

#### Distal neuropathy assessment

A Semmes Weinstein monofilament (SWM) 5.07 was used to evaluate sensory neuropathy in the lower extremities. For SWM 5.07 10 g of force was applied to evaluate the loss of protective sensation in the hand or foot. In this study, 9 test sites on the feet were assessed, namely, 3 plantar sites on each foot, the hallux, third toe, and fifth toe; the bases of the first, third, and fifth metatarsals, the heel, and 2 central sites. Each test site was assessed in triplicate in a blinded and arrhythmic manner. A correct perception of the SWM 2 out of 3 times at each site was given a score of 1, while a lack of perception less than 1 out of 3 times was given a score of 0. The total peripheral neuropathy score was calculated by adding the sum for each foot, and normal sensory function was defined as a total of at least 6 out of 9 sites, while a score less than 5 was considered to indicate the presence of peripheral neuropathy. Areas with ulcers, calluses, necrotic tissue, and scars were avoided during the test.

#### Deep tendon reflexes

The patellar and Achilles reflexes were assessed using the knee and foot jerk tests. A patellar hammer was used to lightly tap the patellar tendon and Achilles tendon, on a relaxed leg. A normal reaction was defined as extension of the lower leg or the foot toward the plantar surface, respectively. Each test was conducted in duplicate on each foot. Reflexes were graded as 0 (Absent), 1 + (Hypoactive), 2 (Normal), 3 (Hyperactive without involuntary muscle contraction) and, 4 + (Hyperactive with involuntary muscle contraction). Only 2 + was considered normal in our study and the absence or hyperactivity of reflexes in at least one lower limb was noted as abnormal.

#### Peripheral vascular disease

Pulses in the dorsalis pedis and posterior tibialis were also recorded and assessed for rate, rhythm, and amplitude using the SonoTrax vascular Doppler ultrasound (EdanUSA, San Diego, California, USA). An amplitude of 3 + was considered to indicate a normal pulse at each test site, and a pulse amplitude less than 2.9 was considered abnormal. Peripheral vascular disease (PVD) was assessed by calculating the ratio of systolic blood pressure in the dorsalis pedis to that in the posterior tibialis, and PVD was defined as a ratio of < 0.9, while a range of 0.9–1 was considered normal function.

#### Ankle-brachial index

The ABI was calculated using the ratio of ankle blood pressure to arm (brachial) blood pressure. An ABI of 0.9–1 was considered normal and indicated the absence of a PVD, while an ABI < 0.9 signified ABI and > 1.2 were excluded due to potential arterial stiffness.

### Self-care deficit assessment

Participants were assessed for self-care deficit by checking whether they (i) were able to see the bottom of their feet, (ii) wore poor fitting footwear, (iii) had not received prior foot care education, (iv) had a foot ulcer but had not reported foot problems to the healthcare provider, or (v) did not take steps to reduce the risk of injury. Self-care deficit was defined by a ‘yes’ answer to any of the following questions.

### Diabetic foot risk scoring

The risk of developing DF ulcers within two years was calculated using the clinical prediction rule (CPR) for diabetic foot ulceration. The scoring parameters considered three fundamentals, namely, sensitivity to a 10 g monofilament, absence of pedal pulses and history of ulceration/amputation. The risk score was then classified using the precise CPR scoring parameters and: sensitivity to 10 g monofilament = 1 point, absence of one pedal pulse = 1 point, and history of ulceration or amputation = 2 points. The total risk was then calculated as the sum of the CPR score, and extrapolated from the risk table.

### Knowledge, attitudes and practices assessment

At recruitment, a semistructured questionnaire was administered to participants to assess their knowledge, attitudes, and practices (KAP) related to DF. The questions asked about the frequency of patient foot examination, foot inspection and care in the presence of callosities, cuts and wounds, and foot washing, as previously recommended by the American College of Foot and Ankle Surgeons as well as the Diabetes UK guidelines [20, 21]. The questionnaire was composed of a knowledge section comprising 5 multiple-choice questions with yes, no or unsure responses as the selected responses. The attitudes and practices section included 6 short answer questions. The survey instrument included questions about diabetes medications used to prevent complications, wound management and behavioural factors such as smoking affecting DF risk. To minimize the guessing effect, an unsure response was added to each question. Incorrect or unsure responses were given a score of 0, while the correct responses were assigned a score value of 1. The maximum total KAP score was 11 and a higher score implied better knowledge of diabetes and DF. The KAP scores were then defined as good or poor based on Bloom’s cut-off point [22]. Therefore, knowledge scores > 60% (*i.e.* at least 3 out of 5 correct responses) were regarded as indicating good knowledge. Scores of > 80% *(i.e.* at least 5 out of 6 correct responses) in the attitude and practice section indicated good attitudes/practice, 60–79% (*i.e.,* 4 out of 6 correct responses) indicated moderate attitudes/practices, and < 59% (< 3 out of 6 correct responses) indicated poor attitudes/practices. The questionnaire was made available in the three main languages spoken in Zimbabwe – English, Shona and Ndebele.

### Data analysis

All the data were captured into RedCap^R^ and analysed using STATA version 12 (StataCorp LLC, Station College, TX, USA). Continuous data are summarised as mean ± standard deviation or median (interquartile ranges) while categorical data were expressed as frequencies and percentages. The Chi-square goodness of fit test was used to test for associations between categorical variables. Univariate and multivariate logistic regression analyses were used to evaluate associations analyses between risk factors and DFU or amputation. A threshold of *p* < 0.05 was considered to indicate statistical significance in this study.

## Results

This cohort included 352 participants with a confirmed diagnosis of diabetes (Table 1). The combined mean age was 57.9 ± 14 years. There was no significant difference in age between males and females (*p* = 0.945). Oral hypoglycaemics were the most common treatment for both men (67%) and women (74%). A total of 44 (12.5%) smokers and 15 (4%) alcohol consumers were recorded and the number of men who smoked (*p* < 0.001) and consumed alcohol (*p* = 0.006) was significantly greater than that of women (Table 1). Glycated haemoglobin (HbA1c) values obtained from patient records were available for 35 (10%) participants. The mean HbA1c was 7.9% ± 2.6. Normal A1c levels (< 5.6%) were detected in 20% (*n* = 7) of the participants, while 2.9% (*n* = 1) were considered to have levels that were classified as pre-diabetic (5.7% < A1c < 6.4%), indicating good glycemic control, and 71% (*n* = 27) had HbA1c levels that are reflective of diabetes (A1c > 6.5%).

**Table 1:** Characteristics for *n* = 352 PLWD recruited in Harare, Zimbabwe, between February 2015 – February 2016

**Characteristic**	**Male (*n* = 82)** **n (%)**	**Female (*n* = 270)** **n (%)**	***p*-value**
**Mean Age ± SD (years)** min–max	57 ± 17 14–88	58 ± 13 12–86	0.945
**BMI (kg/m**^**2**^**)**			**0.044**
Underweight (< 18.5)	14 (17)	31 (11)	
Normal (18.6 – 24.9)	30 (37)	77 (29)	
Overweight (25.0–29.9)	22 (26)	99 (37)	
Obese (> 30.0)	16 (20)	63 (23)	
**Diabetes type**			** < 0.001**
I	21 (26)	41 (15)	
II	61 (74)	229 (85)	
**Diabetes duration (years**)			0.820
< 1	21 (26)	50 (19)	
1.1–5	28 (34)	90 (33)	
6–10	12 (15)	46 (17)	
11–15	9 (10)	45 (17)	
> 16	12 (15)	39 (14)	
**Type of treatment**^**a**^			0.533
Oral hypoglycaemics	55 (67)	199 (74)	
Insulin	30 (37)	59 (22)	
Diet	5 (6)	23 (9)	
**Smoking**			** < 0.01**
Never smoked	44 (51)	217 (80)	
Past smoker	22 (25)	30 (11)	
Current smoker	21 (24)	23 (9)	
**Number of cigarettes per day (*n* = 44)**			0.614
< 5	13 (62)	15 (65)	
6–10	7 (33)	8 (35)	
20	1 (5)	0 (0)	
**Alcohol consumption history (*n* = 15)**	10 (12)	5 (2)	**0.006**

^a^Some patients were receiving two or more modalities

### Prevalence of DF complications

DF complications were detected in 53% (95% confidence intervals = 50–56) of the participants. Foot examinations were also conducted, and 33 (9%) participants had discoloured toenails, with fungi (*n* = 13), and fissures (*n* = 19) or low temperature (22%; *n* = 80) (Table 2). Half of the participants presented with distal peripheral neuropathy. Fifty-nine participants (17%) presented with diabetic foot ulcers; 16 were men and 43 were women. The distribution of foot ulcers was comparable between men and women (*p* = 0.451). Lower limb amputations were observed in 7 (3%) participants, more of whom were males (*n* = 4) than women (*n* = 3) (*p* = 0.03).

**Table 2:** Prevalence of factors that have been previously associated with DF among n = 352 participants in this cohort

**Diabetic foot complication**	**Frequency**	
	**n**	**%**
Discoloured toenails	33	9
Fungal nail	13	4
Abnormal foot skin colour	53	15
Lack of joint flexibility	26	7
Gait instability	27	8
Foot Deformities		
Hallux Valgus	14	5
Flat foot	4	1
Hammer toe	11	3
Calluses	5	1
Fissures	19	5
Distal neuropathy		
Unsteadiness in walking	176	50
Burning, aching pain or tenderness	176	50
Prickling sensation on legs and feet	123	35
Numbness of feet/legs	179	51
Loss of touch sensation in feet	172	50
Ankle Brachial Index (*n* = 276)		
Normal	126	46
Sub-clinical	121	44
Severe	26	9
Foot ulcers	59	17
Amputation	7	3
Abnormal ankle reflex	67	19
Abnormal patellar reflex	17	5

**Fig. 1:**
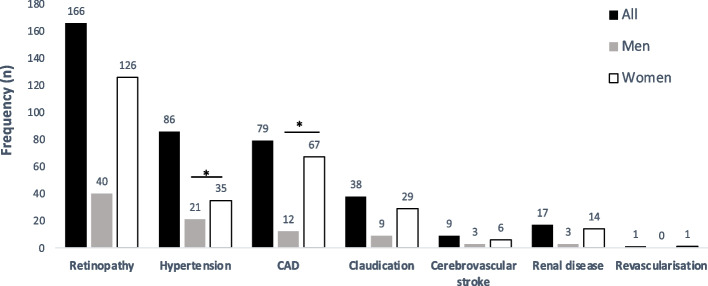
Frequency of complications associated with diabetes complications by sex among *n* = 352 participants. Footnote: * indicates that the frequency difference in frequency between men and women is statistically significant

Among the other diabetes-related complications reported in this cohort, retinopathy (*n* = 166) was the most common complication, followed by hypertension (*n* = 86), coronary artery disease (*n* = 79), claudication (*n* = 38), cerebrovascular stroke (*n* = 9), renal disease (*n* = 17) and revascularization (*n* = 1) (Fig. 1). The frequency of hypertension (*p* = 0.010) and coronary artery disease (CAD) (*p* = 0.048) was significantly greater in women than in men (Fig. 1).

### Risk factors for diabetic foot complications

**Fig. 2:**
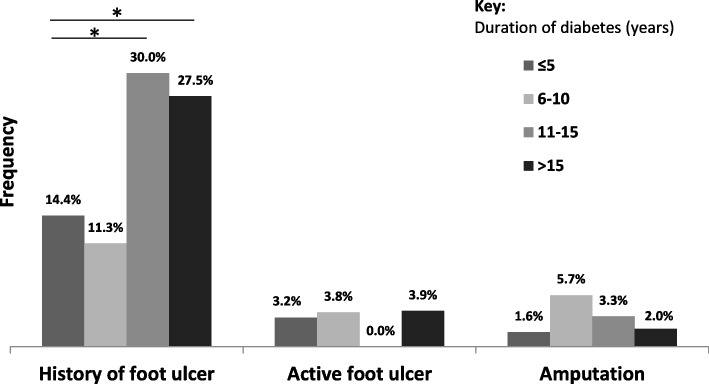
Diabetic foot complication prevalence according to duration of diabetes (*n* = 352). Footnote: * indicates a statistically significant difference (*p* < 0.05)

Analyses for the prevalence of DF complications stratified by the duration of diabetes were also performed (Fig. 2) and a significantly greater frequency of history of foot ulceration was observed for individuals with a long history of diabetes [11–15 years: *p* < 0.001; more than 15 years: *p* < 0.001] was observed.

**Fig. 3:**
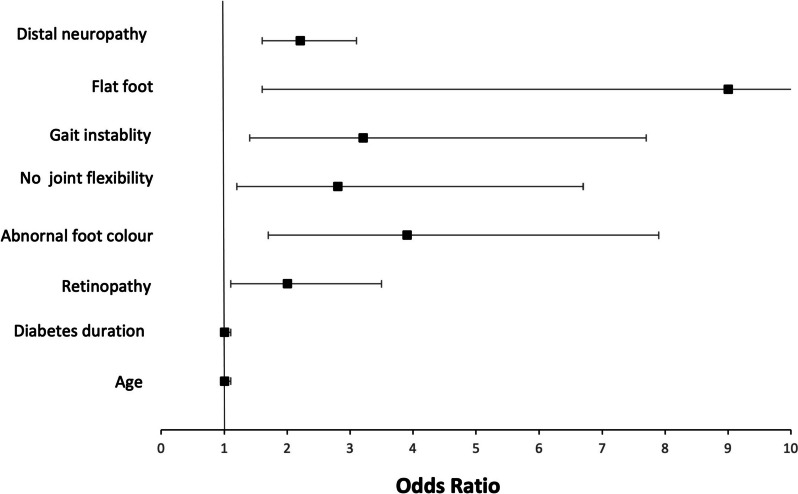
Univariate regression analysis of the factors associated with diabetic foot complications. Footnote: Odds ratios and 95% confidence intervals are shown. Only statistically significant variables (*p* < 0.05) are illustrated in this plot

Univariate regression analysis was performed to identify the risk factors for diabetic complications (Fig. 3). According to the multivariate model, only distal neuropathy (burning, aching pain, or tenderness) was a significant risk factor for diabetic foot ulcers (OR = 1.7; 95% confidence interval = 1.1–2.6; *p* = 0.019) (Table 3). Insulin use was a protective factor against amputation (OR = 0.1; 95% CI = 0.1–0.9; *p* = 0.049) according to both univariate and multivariate analyses (Table 3).

**Table 3:** Factors associated with diabetic foot ulcers and amputation according to multivariable logistic regression models

**Predictor**	**Covariates**	**Odds ratio (95% CI)**	***p***
**Diabetic Foot Ulcers**	Age	1.0 (1.0–1.1)	0.165
	BMI	1.1 (1.0–1.2)	0.272
	Gender	0.7 (0.2–2.6)	0.564
	Duration of diabetes	1.0 (0.9–1.1)	0.850
	Retinopathy	2.1 (0.7–6.4)	0.198
	Foot colour	5.9 (0.9–37.7)	0.061
	Renal disease	3.4 (0.4–31.7)	0.290
	PAD	1.1 (0.5–2.6)	0.823
	Unsteady walking	0.7 (0.4–1.5)	0.407
	Burning, aching pain or tenderness of feet	1.7 (1.1–2.6)	0.019*
	Prickling sensation	0.9 (0.6–1.4)	0.742
	Numbness of feet	1.1 (0.7–1.7)	0.831
**Amputation**	Age	1.0 (0.9–1.1)	0.917
	Gender	0.6 (0.1–3.9)	0.549
	Type of diabetes	1.5 (0.2–11.8)	0.698
	Insulin use	0.1 (0.1–0.9)	0.049*
	Oral hypoglycemics adherence	0.7 (0.1–5.4)	0.692
	Foot ulcer	5.0 (0.6–40.0)	0.127
	Retinopathy	2.7 (0.3–27.8)	0.393
**Both foot ulceration and amputation**	Age	1.0 (1.0 – 1.1)	0.448
	Gender	0.9 (0.3 – 2.5)	0.864
	Type of diabetes	0.6 (0.1 – 3.2)	0.553
	Duration of diabetes	1.0 (1.0 – 1.1)	0.149
	Renal disease	1.6 (0.3 – 7.1)	0.563
	Unsteady walking	1.3 (0.8 – 2.0)	0.234
	Burning, aching pain or tenderness of feet	1.4 (1.0 – 1.9)	0.061
	Prickling sensation	1.0 (0.7 – 1.4)	0.859
	Numbness of feet	0.9 (0.7 -1.2)	0.497
	Insulin use	0.6 (0.2 – 1.9)	0.403
	Oral hypoglycaemics adherence	1.1 (0.5 – 6.1)	0.383
	Diet	1.4 (0.1 – 13.8)	0.797
	Hypertension	2.1 (0.5 – 8.9)	0.297

^*^Indicates adjusted *p*-value

### Self-care deficit assessment

More than one-third (34%) of the cohort demonstrated a high self-care deficit, while 63% had an intermediate deficit, irrespective of sex (*p* > 0.05) (Table 4). In total, 232 participants (66%) were wearing inappropriate shoes. According to multivariate regression models, self-care deficits were associated with fungal nails (OR = 26.6; 95% CI = 2.5–286.4; *p* = 0.007), peripheral neuropathy (OR = 3.5; 95% CI = 1.1–11.2; *p* = 0.034) and wearing inappropriate footwear (OR = 7.3; 95% CI = 1.6–34.4). Self-care deficit was not a predictor of foot ulceration (OR = 1.5; 95% CI = 0.9–2.5; *p* = 0.166) or amputation (OR = 0.8; 95% CI = 0.2–3.2; *p *= 0.793).

**Table 4:** Frequency, and the factors associated with self-care deficit according to univariable logistic regression analysis (*n* = 352)

	**Frequency n (%)**			**OR (95% CI)**	***p***
	**All**	**Male (*n* = 82)**	**Female (*n* = 270)**		
Self-care deficit					
High	120 (34)	30 (37)	90 (33)	1	ref
Intermediate	221 (63)	49 (60)	172 (64)	0.8 (0.4–1.6)	0.550
Low	11 (3)	3 (3)	8 (3)	0.9 (0.1–7.1)	0.893
PAD*				1.0 (0.6–1.7)	0.900
PN*				3.6 (2.3–5.8)	**< 0.001**
Retinopathy*				1.8 (0.4–8.5)	0.454
Arterial stiffness*				0.1 (0.0–1.3)	0.083
Inappropriate footwear				6.0 (1.5–23.7)	**0.011**
Fungal nail				6.9 (1.3–36.2)	**0.022**

^*^Means Univariate regression analysis of the association with self-care knowledge

### Predicting the risk of developing diabetic foot ulcers

The risk for foot ulceration within 2 years was computed using the clinical prediction rule scoring criteria for diabetic foot ulceration. In total, 288 participants had complete data for risk scoring, and 31% exhibited a low risk of foot ulceration (risk = 2.4%; 95% CI = 1.4–3.9%) within 2 years. Six participants (2.1%) had a 51% risk (95% CI = 38—64%) of developing foot ulcers within two years (Table 5).

**Table 5:** Frequency of clinical prediction risk (CPR) scoring for diabetic foot ulcers within two years (*n* = 288)

**Score**	**Risk score (95% CI)**	**n (%)**
0	2.4 (1.4 – 3.9)^a^	90 (31.2)
1	6.0 (3.5 – 9.5)^b^	121 (42.0)
2	14 (8.5 – 21.0)^b^	42 (14.6)
3	29 (19—41)^b^	29 (10.1)
4	51 (38—64)^b^	6 (2.1)

^a^Low risk of foot ulceration within 2 years that does not require intervention

^b^High risk of foot ulceration within 2 years that requires intervention

### Assessment of knowledge of DF

Most of the participants in this study correctly responded that regular medication was important for preventing diabetes-related complications, and foot care was important for preventing injuries, wounding, infections, and ulceration (Table 6). However, more than 96% of the participants did not know that smoking can exacerbate DF.

**Table 6:** Responses to knowledge-related questions among the *n* = 290 participants

**Question**	**n (%)**
**DM patients should take medication regularly to prevent DM complications?**	
Yes	263 (90.7)
No	4 (1.4)
Unsure	23 (7.9)
**DM patients should look after their feet because they may not feel a minor injury to their feet?**	
Yes	248 (85.5)
No	6 (2.1)
Unsure	36 (12.4)
**DM patients should look after their feet because wounds and infection may not heal quickly?**	
Yes	267 (92.0)
No	7 (2.1)
Unsure	17 (5.9)
**DM patients should look after their feet because they may get a foot ulcer?**	
Yes	226 (77.9)
No	18 (6.2)
Unsure	46 (15.9)
**DM patients should not smoke because smoking causes poor circulation and increases risk of diabetic foot?**	
Yes	9 (3.1)
No	51 (17.6)
Unsure	230 (79.3)

### Attitudes and practice assessment

The frequency of correct responses for attitude and practice–related factors is illustrated in Table 7. More than half of the participants were either unsure (37%) or incorrectly responded (~ 18%) to finding redness/blood between toes. In total, 18% (*n* = 52) said they would apply home remedies such as methylated spirit, petroleum jelly products, Eucalyptus oil-based vapor-rub *e.g.* Vicks VaporRub, astringent baby powder, povidone iodine e.g. Betadine, table salt or crushed paracetamol.

**Table 7:** Responses given for the attitudes and practices section of the questionnaire among *n* = 290 participants

	**n (%)**
**How often do you think you should inspect your feet?**	
Correct response (Daily)	146 (50.3)
Incorrect response	46 (15.9)
Unsure	98 (33.8)
**If you found redness/bleeding between your toes, what is the first thing you do?**	
Correct response (Clean it and seek medical attention)	152 (52.4)
Incorrect response	52 (17.9)
Unsure	86 (29.7)
**What would you do if you had a corn/hard skin lesion?**	
Correct response (Seek medical attention for trimming)	127 (43.8)
Incorrect response	54 (18.6)
Unsure	109 (37.6)
**How often do you think your feet should be washed?**	
Correct response (At least once on a daily basis)	256 (88.3)
Incorrect response	1 (0.3)
Unsure	33 (11.4)
**What temperature of water do you think you should wash your feet in?**	
Correct response (Warm)	228 (78.6)
Incorrect response	30 (10.3)
Unsure	30 (10.3)
**How often do you think you should inspect the inside of your footwear for objects or torn lining?**	
Correct response (Before each wear)	225 (77.6)
Incorrect response	9 (3.1)
Unsure	56 (19.3)

An estimated 38% (*n* = 109) of the respondents answered that in the presence of skin lesions or corns, they were unsure of what to do, while 35 of the 52 incorrect responses were to file or cut off the corn/lesion at home. Six respondents indicated that they would do nothing.

### Knowledge, attitudes and practices related to diabetic foot

The total scores for knowledge, attitudes and practices were calculated using Bloom’s cut off, and approximately 87% of the participants demonstrated high knowledge about risk factors for DF (Table 8). A total of 36% demonstrated poor attitudes toward DF prevention, while ~ 27% had moderate attitudes and practices related to DF prevention.

**Table 8:** Total scores on the KAP questionnaire using Bloom’s cut-off categories (*n* = 299)

**Section**	**Category**	**Score range (Blooms range in %)**	**n (%)**
**Knowledge (out of 5)**	High	3—5 (60—100)	259 (86.6)
	Low	0—2 (0—59)	40 (13.4)
**Attitudes and practices (out of 6)**	High	5 – 6 (80 – 100)	111 (37.1)
	Moderate	4 (60—79)	80 (26.8)
	Poor	0 – 3 (< 59)	108 (36.1)

### Associations of knowledge, attitudes and practices with diabetic foot ulcer risk

There was no association between knowledge (OR = 0.9; 95% CI = 1.7–1.2; *p* = 0.575), or attitudes and practices (OR = 1.0; 95% CI = 0.9–1.2′ 0.968; *p* = 0.968) and an increased risk of developing diabetic foot ulcers in two years.

## Discussion

Complications of diabetes, such as foot ulceration and consequently lower limb amputations can be prevented when detected early. The lack of data on these complications can translate to undermanagement for PLWD. In Zimbabwe, only one known study, conducted in 1961, has reported on the prevalence of foot ulcers among PLWD (1%) [13]. Findings from the present study revealed a significantly greater prevalence of foot ulceration (17%), a trend that is expected given the increasing burden of diabetes in Zimbabwe since 1961 [4, 23].

The prevalence of DFU presented in our study also corresponds with reports from other low- and middle-income countries (LMICs) such as Sudan (18%), Tanzania (15%), India (16%) and Cameroon (12%) [6, 24–27]. Data from LMICs such as Ethiopia (31%) and Jordan (70%) have reported a greater frequency of DFUs, while high-income countries such as the UK and Australia have a lower prevalence of DFUs (< 2.5%) than has been reported in the present study [28–32]. These stark dissimilarities can be ascribed to the availability of podiatric care programs as part of primary care in high-income countries that allow for early detection and treatment of DFUs, whereas such services are not well established in LMICs [31, 32]. In a previous publication, we reported a lack of coordinated programs to effectuate DF care, as well as a lack of chiropody services and podiatric specialists in Zimbabwe [19]. Implementing foot training programs in the Zimbabwean health care system is fundamental to improving foot ulcer case finding, promoting early detection and consequently providing early interventions. An example is the “Step by Step foot” (SbS) program”, which was established to educate healthcare workers on diabetic foot problems and management. The SbS has trained > 300 physicians and paramedics from India, Bangladesh, Sri Lanka, Nepal, and Tanzania, resulting in DF case finding, and a reduced prevalence of foot ulcers and amputation rates [1, 33–35]. It has also been reported that the SbS has trained some healthcare workers from Democratic Republic of Congo, Guinea, Botswana, Malawi, Kenya, Ethiopia, Egypt, Zimbabwe, Nigeria, Pakistan, Saudi Arabia, Barbados, St Lucia, St Maarten, St Kitts and the British Virgin Islands [1, 33–36]. Another similar initiative, the “Train the Foot Trainer” program has also trained healthcare workers from more than 70 LMICs worldwide [36]. A challenge reported by these projects has been the lack of sustainable integration of these programs into the public health systems [1]. The adoption and integration of foot screening programs through the government systems in Zimbabwe could be key to ensuring that DF screening is sustainable in Zimbabwe.

A longer duration of diabetes was associated with an increased risk of diabetic foot ulcers according to the topical data. Our findings are consistent with the literature [37, 38]. A longer duration of diabetes is associated with a higher rate of microvascular and macrovascular complications such as peripheral neuropathy, and cumulative effects of poor glycaemic control [38]. Consistently, the topical data also showed that use of insulin therapy is protective against amputations. Insulin is associated with inflammation reduction, revascularization and wound healing, thereby acting as a protective factor against diabetes-related complications and amputations, in concordance with the topical data [39, 40]. However, for PLWD in Zimbabwe, there can be a disrupted supply chain of essential medications, such as insulin, which can impede adherence, and can be exacerbated for people who have lived with diabetes for longer [4, 16, 41].

In 2022, 44% of the 9 million Zimbabweans of working age were employed, with a wage range of USD100-931 for low- to high-skilled employees [42, 43]. Given the high cost of insulin per month (USD135), affordability is very limited, and there are challenges in subscribing to medical aid which allows people to access subsidised medications and healthcare [42, 43]. Moreover, with these limited funds, the affordability of routine laboratory tests such as HbA1c, and adherence to the specific dietary requirements for glycemic control may also challenge PLWD in Zimbabwe. This is also evidenced by the low number of participants in this study with HbA1c values recorded in their clinical records (10%). Most PLWD monitor their blood glucose levels using the random blood glucose and fasting blood glucose tests. Compared with the blood glucose tests, the HbA1c has been validated to be a more reliable measure of overall glycemic control; however its unaffordability makes this test unpopular in Zimbabwe. This finding highlights the need for subsidised costs for HbA1c monitoring, and diabetic medication for PLWD to improve management and adherence.

This study also reported a high prevalence of peripheral neuropathy (53%) and peripheral arterial disease (PAD) (53%), in agreement with the findings of a previous study from Zimbabwe [18]. Both peripheral neuropathy and PAD are key indicators for foot at risk of DFU, and in the present study, a high prevalence (68%) of foot ulceration risk that requires immediate medical attention was reported. These findings resonate with those of Mukona et. al. [18]. The prevalence of peripheral neuropathy and PAD reported in this study are significantly greater than those recorded in the USA (6% and 9.5% respectively), possibly because of the lack of foot screening services in Zimbabwe [44, 45]. Evidence of the impact of regular foot screening for reducing DF has been widely published, including in an Australian study that confirmed that non-indigenous people who frequently visited the clinic and received regular foot screening had a lower prevalence of DF than indigenous people who had poor health-seeking behaviours and limited resources in their community health facilities [30, 46].

Taken together, these findings emphasise the urgent need for regular foot screening as an integral part of care for PLWD in Zimbabwe. However, although published data highlight the urgent need for DF services in Zimbabwe, such services are not yet available due to a lack of coordinated guidelines for foot screening and limited resources or manpower for podiatric care [4, 16, 19]. Due to these health system-related challenges in screening and managing DF, it is equally vital to engage patients as a part of the holistic diabetes care journey, not only in glycaemic control but also in conducting self-screening for complications such as foot care.

From the current data, good knowledge of DF was demonstrated by the participants, however, poor attitudes and practices toward DF were found. Correspondingly, > 90% of participants were found to have DF self-care deficit. It is likely that although participants may know practices that increase the risk of DF, poor attitudes and may be ascribable to the low socioeconomic background of the cohort, that may hinder the recommended practices that can prevent DF.

Moreover, our study showed that 97% of respondents did not know that smoking can affect blood circulation, and therefore increase the risk of DF. This finding demonstrates the need to emphasise the role of behavioural risk factors such as smoking in diabetes-related complications and to further the understanding of why PLWD need to modify their lifestyle and improve their diet. Similar programs have been implemented, at no cost to the patient, in the United Kingdom to encourage PLWD to take an active role in diabetes management [47–49]. An example is the Dose Adjustment For Normal Eating (DAFNE) course, a program that has been administered to PLWD in the UK since 2002, and has improved the quality of life, blood-glucose control, and reduced diabetes-related hospitalisation [49, 50]. With adequate resources and political will, programs such as DAFNE can be tailored to the socioeconomic conditions and adopted in Zimbabwe as a way to educate PLWD. In addition, there are also mobile health tools that have been developed to promote foot care for PLWD, such as the MyFootCare mobile application in Australia [51]. The MyFootCare promotes self-monitoring of ulcers, helps individuals identify precursors of DF, drives foot care monitoring and promotes general self-care [51]. Therefore, such mobile-health tools can be adopted in Zimbabwe for PLWD with smartphones, as an additional part of the educational and awareness package.

This study is limited in that the questionnaire used combined attitudes and practices. In the future, a more comprehensive questionnaire that independently addresses knowledge, attitudes, practices and perception questions of DF care will be used. This approach is fundamental towards accurately revealing misconceptions about footcare and developing relevant educational and awareness tools for PLWD in Zimbabwe.

## Conclusion

This study revealed a high prevalence of diabetic foot ulcers among PLWD in Harare, Zimbabwe; and that distal peripheral neuropathy was a major risk factor for foot ulceration. These findings indicate the need for regular foot screening as an integral part of primary care. This study also reports on the protective role of insulin against amputations, underscoring the need to increase insulin administration in PLWD, to prevent diabetes-related complications. Taken together, our study, and the previous studies on diabetes in Zimbabwe highlight the need for policies that drive equitable access to diabetes medications such as insulin as well as screening facilities for DF, as a step toward preventing diabetes-related complications *e.g.,* DF. This study also reported on poor attitudes and practices related to DF, corresponding with the self-care deficit which was also demonstrated in this cohort. This highlights the need for education and training initiatives to be established for PLWD to promote self-care, as a key prevention tool against DF.

## Data Availability

The datasets used and/or analysed during the current study are available from the corresponding author upon reasonable request.
